# Relationship between Meditative Practice and Self-Reported Mindfulness: The MINDSENS Composite Index

**DOI:** 10.1371/journal.pone.0086622

**Published:** 2014-01-22

**Authors:** Joaquim Soler, Ausiàs Cebolla, Albert Feliu-Soler, Marcelo M. P. Demarzo, Juan C. Pascual, Rosa Baños, Javier García-Campayo

**Affiliations:** 1 Department of Psychiatry, Hospital de la Santa Creu i Sant Pau, Barcelona, Spain; 2 Universitat Autònoma de Barcelona (UAB), Centro de Investigación Biomédica en Red de Salud Mental (CIBERSAM), Institut d’Investigació Biomèdica- Sant Pau (IIB-SANT PAU), Barcelona, Spain; 3 Ciber Fisiopatologia de la obesidad y la nutrición (CIBEROBN) Santiago de Compostela, Spain; 4 Departament de psicologia bàsica, clínica i psicobiologia, Universitat Jaume I, Castelló, Spain; 5 “Mente Aberta” – Brazilian Center for Mindfulness and Health Promotion. Department of Preventive Medicine, Federal University of Sao Paulo (UNIFESP), Sao Paulo, Brazil; 6 Departament de Personalitat, Avaluació i Tractaments Psicològics, University of Valencia, Valencia, Spain; 7 Department of Psychiatry, Miguel Servet Hospital & University of Zaragoza, Instituto Aragonés de Ciencias de la Salud, Red de Actividades Preventivas y de Promoción de la Salud (REDIAPP), Zaragoza, Spain; Penn State University, United States of America

## Abstract

Mindfulness has been described as an inherent human capability that can be learned and trained, and its improvement has been associated with better health outcomes in both medicine and psychology. Although the role of practice is central to most mindfulness programs, practice-related improvements in mindfulness skills is not consistently reported and little is known about how the characteristics of meditative practice affect different components of mindfulness. The present study explores the role of practice parameters on self-reported mindfulness skills. A total of 670 voluntary participants with and without previous meditation experience (n = 384 and n = 286, respectively) responded to an internet-based survey on various aspects of their meditative practice (type of meditation, length of session, frequency, and lifetime practice). Participants also completed the Five Facets Mindfulness Questionnaire (FFMQ), and the Experiences Questionnaire (EQ). The group with meditation experience obtained significantly higher scores on all facets of FFMQ and EQ questionnaires compared to the group without experience. However different effect sizes were observed, with stronger effects for the *Observing* and *Non-Reactivity* facets of the FFMQ, moderate effects for *Decentering* in EQ, and a weak effect for *Non-judging*, *Describing*, and *Acting with awareness* on the FFMQ. Our results indicate that not all practice variables are equally relevant in terms of developing mindfulness skills. Frequency and lifetime practice – but not session length or meditation type – were associated with higher mindfulness skills. Given that these 6 mindfulness aspects show variable sensitivity to practice, we created a composite index (MINDSENS) consisting of those items from FFMQ and EQ that showed the strongest response to practice. The MINDSENS index was able to correctly discriminate daily meditators from non-meditators in 82.3% of cases. These findings may contribute to the understanding of the development of mindfulness skills and support trainers and researchers in improving mindfulness-oriented practices and programs.

## Introduction

A decade after the first meta-analysis showing the beneficial effects of mindfulness on health was published [1], the body of evidence on the benefits of mindfulness practice to treat stress, anxiety, and depressive symptoms continues to grow. Mindfulness has been proven effective not only for the general population, but also to treat several specific clinical populations [2], [3], [4], [5].

According to the traditional roots of mindfulness, it is assumed that long-term meditation practice cultivates mindfulness skills and that development of such skills, in turn, promote psychological well-being [6]. From this perspective, several authors have described mindfulness as an inherent capability that can be learned and practiced by everyone [7], [8], [9]. Studies that have evaluated the inherent ability to achieve mindfulness in meditation-naive subjects have found an association between mindfulness trait scores and broad indexes of physical and mental health and overall well-being [1], [10]. Furthermore, the role of regular meditative practice seems to be highly relevant to outcomes. For this reason, most mindfulness programs teach a wide range of practices and require home-practice to improve mindfulness skills. In Mindfulness Based Stress Reduction program (MBSR) and Mindfulness Based Cognitive Therapy (MBCT), 45 minutes of daily practice is the standard [8], [11]. Regular practice of mindfulness meditation seems to be essential to attain the therapeutic benefits of mindfulness-based programs. However, the optimum balance between the dose of meditative practice and clinically-relevant health outcomes is still not clear. One important area of current mindfulness research is to determine this optimal practice time in order to design more effective and cost-effective mindfulness programs and training.

In one of the most remarkable studies on the effects of mindfulness practice [12], 174 subjects participated in an 8-week MBSR program. The aim of the study was to evaluate changes in participants’ pre- and post-mindfulness training stress levels, medical symptoms, anxiety, pain, and mindfulness skills. Results from that study showed that the amount of time spent on mindfulness practice at home was significantly associated with changes in specific components of the Five Facets Mindfulness Questionnaire (FFMQ) [13], thus demonstrating that the amount of “formal practice” (i.e., sitting meditation, body scan, yoga) were associated with an increase in the subject’s ability to not react immediately to an inner experience and, to a lesser extent, with the capacity to observe mindfully both internal and external experiences, and to act with awareness during daily activities, rather than behaving in an “autopilot” mode. In another study, 83 chronically-ill patients who participated in a MBSR program [14] were evaluated to determine the relationship between varying doses of mindfulness exercises, mindfulness skills development (assessed with the Mindfulness Attention Awareness Scale or MAAS; [10]), and certain health indices. Surprisingly, neither the type nor the dose of formal mindfulness practice was associated with clinical outcomes or increases in self-reported mindfulness scores. In fact, only one type of informal practice (breath awareness) was found to increase mindfulness skills and to reduce clinical symptoms. Notwithstanding these findings, it is important to point out that this was a non-follow-up study, and typically more than 8 weeks of stable meditative practice is needed to achieve significant results.

One review assessed the association between amount of practice, measures of mindfulness skills, and clinically-relevant outcomes [15]. In that review, the authors found no clear evidence of a straightforward relationship between these variables and outcomes. This lack of consistency across studies regarding the influence of mindfulness practice on outcomes might be attributable to several problems, including a lack of a consensus definition of mindfulness practice, non-validated methods of measuring the amount of practice, and/or variations across study populations [14]. In addition, as several authors have pointed out [6], [16], [17], [18], measuring mindfulness is difficult and there is no “gold-standard” measure. There are other important issues that still need to be resolved, including the need to clarify the relationship between state and trait mindfulness, and to minimize the idiosyncratic interpretability of items that might not actually reflect the subject’s knowledge of and experience with mindfulness. A final, unresolved issue is the lack of an instrument to reliably assess how various amounts of practice affects outcomes. In this regard, Grossman [17] suggested that the relationship between self-reported mindfulness skills and mindfulness practice should be studied to ascertain whether pragmatic meditation variables–such as type of mediation, amount of daily practice, or years of experience– actually yield different scores on questionnaires designed to measure mindfulness.

Several studies have compared self-reported mindfulness in large samples of meditators and non-meditators [19], [6], [20], [21], [22], [23]. In general, these studies have found that while certain aspects of mindfulness do correlate coherently with the amount of meditative practice and are associated with improvements in well-being, other facets seem to have an erratic and anomalous pattern across different studies and populations. For example, one anomaly is the positive correlation between Observing and psychopathological symptoms found in one study [13]. Given these varied findings, it seems clear that mindfulness is a highly complex skill that is challenging to measure, in part because it is sensitive to prior meditation experience and to different methodological approaches [23], [24], [25].

Most previous studies have focused primarily on validating scales and/or on how the psychometric aspects of the various facets of mindfulness vary in response to the target population. In contrast, few studies have focused on analyzing in detail the pragmatic variables of meditative practice. Given the relative paucity of such studies, the present study was designed to explore whether certain dose-related variables of meditative practice (type of meditation, length of session, frequency, and lifetime practice) predict self-reported mindfulness skills in a broad sample of meditators and non-meditators.

## Method

### Design of the Study

This was an observational, cross-sectional study.

### Participants and Data Collection

A survey containing several questionnaires was developed using a commercial online survey system (www.surveymonkey.com; Portland, OR, USA), and a link to this website was posted on several Spanish scientific research portals involved in mindfulness and meditation research. It was also sent to several mindfulness associations, Zen monasteries, and *sanghas*, and to a non-meditator convenience sample. The survey was available for response between April 2011 and December 2012. Although this was an online survey, previous studies have shown that data collected from the Internet is as reliable as traditional paper-pencil methods [26].

A total of 917 subjects accessed the link, and 850 voluntarily agreed to participate; of these, 670 fully completed all the questions and scales of the survey. Based on self-reported previous meditative experience, the sample was classified into a Meditator group (MG; n = 384) and a Non-meditator group (NMG; n = 286). Table 1 shows all relevant sociodemographic data. The study was approved by the Aragon Ethics Committee and performed in accordance with the ethical standards of the 1964 Declaration of Helsinki. All participants gave written informed consent prior to inclusion in the study.

**Table 1:** Description of the samples with and without meditative experience.

	MG (N = 384)	NMG (N = 286)	*p*
**Sex (female/%)**	213/55.47%	203/70.98%	<0.001
**Age**	43.9 (10.64)	37.85 (10.85)	<0.001
**Years of schooling**	16.36 (2.89)	16.15 (2.76)	*n.s.*

Footnote: Percentage (%) or mean scores (SD) are represented when appropriate. T-tests were used for quantitative variables and chi-square for Sex. MG =  Meditator group; NMG = Non-meditator group.

### Meditative Practice Variables

Participants were asked about characteristics of their practice, specifically to provide: 1) the type of meditation they practiced (e.g. Mindfulness/Vipassana, Zen, Tibetan, yoga) – *type*; 2) the average length of their meditation sessions (minutes per session) – *session length*; 3) how often they practiced in terms of days per month – *frequency*; and 4) how long they had been practicing (in months) – *lifetime practice*. These various practice parameters were used to assess the type and dose of practice and their association with various facets of mindfulness.

### Instruments

The FFMQ [13]: This questionnaire consists of 39 items that assess five facets of mindfulness. Items are rated on a Likert scale ranging from 1 (never or very rarely true) to 5 (very often or always true), with higher scores meaning higher self-reported mindfulness skills. The five facets are as follows: *Observing*, which involves noticing or attending to internal and external experiences such as sensations, thoughts, or emotions; *Describing,* which refers to labelling internal experiences with words; *Acting with awareness,* which involves focusing on one’s activities at a given moment as opposed to behaving mechanically; *Non-judging of inner experience*, which refers to taking a non-evaluative stance toward thoughts and feelings; and *Non-reactivity to inner experience*, which is defined as allowing thoughts and feelings to come and go, without getting caught up in or carried away by them. The Spanish version of the FFMQ has been validated and shows good psychometric properties (with Cronbach’s alpha scores ranging from 0.8 to 0.91; [20]).

Experiences Questionnaire (EQ) [27]: this 11-item questionnaire was designed to measure the capacity to observe one’s thoughts and feelings as temporary and objective events of the mind, also known as *Decentering*, which is a key element of mindfulness training. Items from the scale are rated on a Likert scale ranging from 1 (never or very rarely true) to 5 (very often or always true), with higher scores indicating greater “decentering”. EQ also has been shown to have good psychometric properties (Cronbach’s alpha = 0.90) [27].

### Data Analysis

T-tests and chi-square tests were used to assess socio-demographic differences between MG and NMG. A two-way MANCOVA was performed to assess whether there were differences in self-reported mindfulness scores. The MANCOVA include Group and Sex as factors, FFMQ and EQ subscales as dependent variables, and Age as a co-variable. MANOVA was used to determine differences in the scoring of mindfulness facets between different types of meditation practices (e.g. Zen, Mindfulness/Vipassana and other practices) using FFMQ and EQ scores within the MG.

Linear regression (LR) analyses were performed for all mindfulness variables (i.e., each FFMQ subscale and the overall EQ score) as dependent variables and measures of meditative practice (i.e., Total months of practice, Total days of practice per month, Average length of a meditation session) and possible confounding variables (such as Age, Years of schooling, and Sex) as independent variables. Practice variables were evaluated to check for co-linearity. All practice variables for participants without meditative experience were assumed to be zero.

To select the items most closely associated with meditative practice, all items from the FFMQ and EQ and practice measures were standardized and entered into an Exploratory Factorial Analysis (EFA). All items (n = 19) which were allocated in the same factor of meditation practice measurements were used to create the new collection of items considered sensitive to mindfulness practice (the 19-item composite index was designated “MINDSENS”). A total MINDSENS score was obtained by averaging the scores on all 19 items. A two-way MANCOVA was performed to assess between-group differences in MINDSENS scores. Subsequently, a stepwise discriminant analysis was performed to test whether MINDSENS discriminated between daily practitioners and non-meditators.

## Results

### Meditative Practice Variables

The types of meditation practiced by participants in the MG were as follows: Zen (37%), Mindfulness/Vipassana (49%), or other types of meditation (e.g., Yoga, Tibetan), (14%). No significant differences between practice type and FFMQ and EQ scales were observed. The amount of meditative practice in MG was as follows: total months of previous meditation practice ranged from 0.5 to 516 months (mean = 85.76; standard deviation [SD] = 95.89), days of practice per month ranged from 1 to 28 (mean = 14.71; SD = 10.91) and minutes per session ranged from 7 to 120 (mean = 33.71; SD = 19.3).

### Differences in Self-reported Mindfulness Skills between Groups with and without Meditative Experience

After controlling by sex and age, significant differences were observed between MG and NMG groups on all FFMQ and EQ subscales, with effect sizes ranging from high to low, depending on the subscale (see Table 2 for more detailed information).

**Table 2:** Differences between the meditator and non-meditator groups in mindfulness facets.

	MG	NMG	Univariate	*d*
**FFMQ-Observing**	30.44 (4.71)	25.59 (5.48)	<0.001	0.95
**FFMQ-Describing**	30.47 (5.3)	29.31 (5.98)	0.03	0.20
**FFMQ-Awareness**	27.34 (5.14)	26.02 (5.65)	0.043	0.24
**FFMQ-Non-judging**	30.61 (6.5)	27.55 (6.88)	<0.001	0.46
**FFMQ-Non-reactivity**	24.84 (4.14)	21.22 (4.41)	<0.001	0.85
**EQ-Decentering**	41.07 (6.07)	36.61 (6.32)	<0.001	0.72

Footnote: Mean scores with standard deviations (SD), univariate analyses and Cohen’s *d* are represented. Group and sex were introduced as factors, and age as a co-variable in a two-way MANCOVA analysis with scores in FFMQ subscales and EQ as a dependent variables. Significant effects were observed for group (p<0.001), age (p<0.001), and sex (p = 0.028). Non-interactive Group × Age effect was observed (p>0.5). No differences were observed regarding mindfulness self-reported scores in FFMQ subscales and EQ between the different types of meditation (all p>0.05). MG =  Meditator group; NMG = Non-meditator group.

### The Role of Meditative Practice on Self-reported Mindfulness Variables

Table 3 provides a summary of all regression analyses for all FFMQ and EQ mindfulness subscales. Independent variables were as follows: total months of practice; total days of practice per month; average length of a meditation session; age; years of schooling; and sex.

**Table 3:** Linear regression models for self-reported mindfulness subscales according to meditative practice and confounding variables.

	Regressionmodel	Components					
		Age	Sex	Years ofschooling	Days permonth	Months ofpractice	Length ofmeditationsession
**FFMQ Observing**	R^2^ = 0.214p<0.001	β = − 0.83p = 0.038	*n.s.*	*n.s.*	β = 0.278p<0.001	β = 0.174p<0.001	β = 0.147p = 0.002
**FFMQ Describing**	R^2^ = 0.053p<0.001	*n.s.*	*n.s.*	β = 0.176p<0.001	*n.s.*	*n.s.*	*n.s.*
**FFMQ Awareness**	R^2^ = 0.059p<0.001	*n.s.*	*n.s.*	*n.s.*	β = 0.145p = 0.006	β = 0.130p = 0.006	*n.s.*
**FFMQ Non-judging**	R^2^ = 0.093p<0.001	*n.s.*	*n.s.*	β = 0.121p = 0.002	β = 0.180p<0.001	β = 0.116p = 0.012	*n.s.*
**FFMQ Non-reacting**	R^2^ = 0.22p<0.001	*n.s.*	β = 0.104p = 0.006	β = 0.120p = 0.001	β = 0.197p<0.001	β = 0.202p<0.001	*n.s.*
**EQ Decentering**	R^2^ = 0.211p<0.001	β = 0.086p = 0.038	*n.s.*	β = 0.130p = 0.001	β = 0.292p<0.001	β = 0.137p = 0.002	*n.s.*

Footnote: FFMQ =  Five Facets Mindfulness Questionnaire; EQ =  Experiences Questionnaire.

### Creation of MINDSENS Index

In order to select the FFMQ and EQ items that were most closely related to meditative practice, we performed an EFA on all standardized values obtained from the FFMQ and EQ survey questions, and on the three parameters that assessed meditative dose (i.e. total months of practice, total days of practice per month, and average length of a meditation session). The Keiser-Mayer-Olkin index was 0.944 and the Bartlett’s test was significant (p<0.001), and the first rotated factor (which explained 28% of the variance) included the three measurements of meditative practice and 19 items from the FFMQ (items 1, 29, 33, 19, 21, 36, 26, 20, 24, and 31) and the EQ (items 9, 4, 2, 3, 10, 11, 5, 7, and 8). See Table 4 for more details on specific items and factorial loads.

**Table 4:** Rotated factorial loads of standardized FFMQ and EQ items and meditative practice variables.

Variable	Factorial load
PRACTICE: Total days of practice per month	0.64
PRACTICE: Average length of a meditation session	0.59
PRACTICE: Total months of meditative practice	0.53
FFMQ-1- Observing -*When I’m walking, I deliberately notice the sensations of my body moving.*	0.62
FFMQ-36 Observing - *I pay attention to how my emotions affect my thoughts and behaviour.*	0.51
FFMQ-26- Observing - *I notice the smells and aromas of things.*	0.48
FFMQ-20- Observing - *I pay attention to sounds, such as clocks ticking, birds chirping, or cars passing.*	0.48
FFMQ-31- Observing - *I notice visual elements in art or nature, such as colours, shapes, textures, or patterns of light and shadow.*	0.43
FFMQ-29- Non-reacting - *When I have distressing thoughts or images I am able just to notice them without reacting.*	0.62
FFMQ-33- Non-reacting -*When I have distressing thoughts or images, I just notice them and let them go.*	0.59
FFMQ-19- Non-reacting - *When I have distressing thoughts or images, I “step back” and am aware of the thought or image without getting* *taken over by it.*	0.59
FFMQ-21- Non-reacting - *In difficult situations, I can pause without immediately reacting.*	0.55
FFMQ-24- Non-reacting - *When I have distressing thoughts or images, I feel calm soon after.*	0.46
EQ-9 - *I can actually see that I am not my thoughts.*	0.56
EQ-4 - *I can separate myself from my thoughts and feelings.*	0.56
EQ-2 - *I can slow my thinking at times of stress.*	0.53
EQ-3 - *I notice that I don’t take difficulties so personally.*	0.52
EQ-10 - *I am consciously aware of a sense of my body as a whole.*	0.50
EQ-11 - *I view things from a wider perspective.*	0.49
EQ-5 - *I can take time to respond to difficulties.*	0.48
EQ-7 - *I can observe unpleasant feelings without being drawn into them.*	0.47
EQ-8 - *I have the sense that I am fully aware of what is going on around me and inside me.*	0.36

Footnote: Scale, item number and the specific subscale to which the item belongs were represented when appropriate. Item content appears in cursive. FFMQ =  Five Facets Mindfulness Questionnaire; EQ =  Experiences Questionnaire.

The role of meditative practice in MINDSENS scoring was studied by means of an ulterior LR that revealed a significant model (R2 = 0.277; p<0.001) which included years of schooling (β = 0.110; p = 0.003), frequency (β = 0.309; p<0.001), lifetime practice (β = 0.179; p<0.001), and session length (β = 0.1; p = 0.032) as independent variables. A two-way MANCOVA with MINDSENS as a dependent variable indicated a significant between-groups difference in MINDSENS scores (MG, 3.70 [0.5]; NMG, 3.20 [0.5]; p<0.001; *d* = 0.95).

Finally a discriminant function analysis was performed to compare the subgroup of daily practitioners (n = 121) with NMG. The stepwise discriminant function analysis with the MINDSENS showed a Wilks’ λ = .610, X2 = 194.357 (p<.001). The discriminant function accounted for 100% of the between-group variability. The canonical correlation was.624. The classification ability of the MINDSENS (Table 4) was 82.3% (see Table 5).

**Table 5:** Discriminant analysis of MINDSENS with regard to its ability to detect participants who meditate daily versus those without meditative experience.

Actual Group Membership	Predicted Group Membership with MINDSENS	
	Daily practitioners	NMG
**Daily practitioners** (N = 121)	103 (85.1%)	18 (14.9%)
**Non-meditators** (N = 275)	52 (18.9%)	223 (81.1%)
	Correctly classified 82.3% of the original group cases	

Footnote: number of cases and percentages (%) are represented. NMG = Non-meditator group.

## Discussion

The purpose of this study was to investigate the relationship between dose of meditation practice and self-reported mindfulness skills. Our results indicated that participants with prior meditation experience reported higher scores on all mindfulness aspects versus meditation-naive participants. *Observing*, *Non-reactivity*, and *Decentering* were the three aspects of Mindfulness that best differentiated between meditators and non-meditators. Furthermore, these three skills were closely associated with frequency and lifetime practice of meditation. Given the specificity that several items from the FFMQ and EQ have for practice variables, we created the MINDSENS composite index. Congruent with previous analyses, MINDSENS included only items from *Observing*, *Non-reactivity* and *Decentering*. This new composite index showed a high capacity to correctly discriminate between daily mediation practitioners and non-meditators. The use of MINDSENS, together with related sociodemographic variables, to explore the role of meditative practice yield data that suggests that frequency of meditation practice, lifetime meditation experience, Years of schooling, and Age were more closely associated with self-reported mindfulness than length of meditative sessions. We observed no differences among meditation types regarding mindfulness skill scores.

### Mindfulness Aspects and Meditative Experience

In agreement with the results reported by previous studies that have evaluated FFMQ scores in samples of meditators and meditation-naive subjects ([6], [21], [22], [23]), meditators scored higher on all mindfulness facets in our study. Effect sizes indicate that two facets (*Observing* and *Non Reactivity*), together with *Decentering* (EQ), are clearly related to previous mediation experience. These findings are congruent with the emphasis that most mindfulness interventions place on homework assignments and with other studies that have reported the same association ([12], [28], [29]). In our study, regression analysis of practice variables showed that the most relevant variables were frequency of practice and lifetime meditation experience. Both of these variables influence 5 out of 6 of the mindfulness facets evaluated. In contrast, meditative session lenght was related only to the development of *Observing*. Despite these findings, it is important to point out that given that *Observing* seems to be an essential and core skill in developing mindful traits, session length may still be an important element in mindfulness-based programs.

We also found that overall length lifetime experience of meditation has an accumulative effect on mindfulness levels. In general, our findings suggest that, to develop mindfulness, it is more useful to meditate for short periods (e.g., 20 minutes/session) on a daily basis rather than to only meditate once a week in a longer session (e.g., 2 hours). It seems likely that frequent practice helps meditators to maintain a mindful stance in everyday life, which is a main goal of mindfulness-based interventions [30]. If this finding is confirmed by further studies, it may enable us to improve adherence in both clinical and non-clinical populations, as shorter sessions will make adherence easier. As with previous studies carried out with experienced meditators [19], [6]. [21], [22], [23], our results suggest that *Observing* may be especially sensitive to previous mindfulness experience. Additionally, several studies performed in non-meditator subjects have failed to establish an association between “Observing” and other facets of mindfulness, or have even found a negative relationship [13], [6], [20] [21], [22], [23]). Taken altogether, these findings support the hypothesis that the practice of mindfulness is closely associated with *Observing* [23] and our results suggest that the ability to observe could be a trainable skill.

Our results showed that *Non-judging* was less closely related to practice than we had initially expected, especially considering that all mindfulness traditions place a great emphasis on developing this attitude [31]. This outcome is also consistent with another study [23] that failed to find the expected connection between *Non-judging* and clusters where meditators were over-represented. As the authors pointed out, the relationship between *Observing* and *Non-judging* was more complex than expected [23]. It seems probable that *Non-judging* is a mindset that can be developed in other ways besides the practice of meditation. For instance, in DBT, not being judgmental is also learned through psychoeducational interventions [30].

It should be mentioned, as Baer et al. [13] pointed out, that *Non-judging* and *Non-reactivity* represent “acceptance” in the FFMQ scale. In our study, *Non-reactivity* was found to be associated with meditation practice. Interestingly, Lilja et al. [23] observed that meditators reported high scores in *Observing* and *Non-reactivity* (independently of *Non-judging* scores). Following this same line of inquiry, Baer et al. [6] performed a mediation analysis that tested facets of mindfulness in relation to meditation experiences, and concluded that *Non-reactivity* (and *Observing*) showed higher beta values, which are indicative of stronger relations. On the whole, these findings seem to indicate that the *Non-reactivity* facet of mindfulness could be a better indication of acceptance in FFMQ than *Non-judging*.

*Decentering* also seems to be relevant to the meditation experience and is sensitive to frequency and lifetime practice. Our study is the first to use the EQ scale (in contrast to the FFMQ) to compare samples with and without meditation experience. Increases in meta-awareness were previously linked to Cognitive Behavioural Therapy (CBT) and also associated with subsequent improvements [32]. This shows that *Decentering* does not seem to be exclusively associated with mindfulness practice and could also be considered a necessary skill for healthy psychological function, as the lack of this skill is believed to be a general vulnerability factor [27].

In our study, *Acting with Awareness* was the only aspect of mindfulness that was exclusively associated (although weakly) with practice-related variables. Nevertheless, low effect sizes were observed when we compared meditators to non-meditators, and practice variables only explained 6% of the variance in the regression model. These findings differed from Baer’s results [6], in which *Acting with Awareness* was related to age and was the only non-significant facet when meditators were compared to non-meditators.

A weak relationship was also found between practice variables and the *Describing* facet. Given that our sample was non-clinical and that most practiced the Zen and mindfulness/Vipassana traditions, these factors may have had an effect on findings related to *Describing*, especially because these traditions generally place little emphasis on the use of verbal labelling in contrast to many mindfulness interventions such as DBT [9] and Acceptance and Commitment Therapy (ACT) [34], which are exclusively designed for clinical populations. Exercises that use verbal labelling for emotions, cognitions, and sensations are commonly used in both DBT and ACT [35]. The role of description in mindfulness is complex: in many practices (e.g., mindfulness of sound) description is to be avoided, whereas it is recommended in others (e.g., mindfulness with emotions) because it helps the practitioner to take a step back and avoid becoming entangled with the experience [36], and it also fosters emotional regulation [37]. In our study, *Describing* is the only mindfulness aspect that showed no association with any of the practice variables, and it had only a slight relation to the variable *Years of schooling*. This result raises the question of whether *Describing* should be considered a feature of mindfulness. *Describing* is completely conceptual in nature and, as Cardaciotto et al. [38] pointed out, while it may be useful, it is not central to mindfulness.

### MINDSENS Composite Index

Given these differences between various aspects of mindfulness, we decided to explore which specific items of the FFMQ and EQ were most sensitive to meditative practice. The aim was to identify those aspects of mindfulness that are potentially trainable and are also further developed by the amount of practice. Once again, we found that the skills *Observing*, *Non-reactivity,* and *Decentering* were the only relevant aspects. This suggests that these are the 3 aspects of mindfulness that are most amenable to improvement through regular practice. It should be pointed out that although EQ measures a global construct, *Decentering* has three different components: the ability to distinguish oneself from one’s thoughts, the ability not to habitually react to one’s negative experience, and the capacity for self-compassion [27]. As a result, there is some overlap between EQ and FFMQ items, especially in *Non-reactivity*. Indeed, this is partially the case with 2 items: Item 5 and 7 (“I can take time to respond to difficulties” and “I can observe unpleasant feelings without being drawn into them”). However, the remaining 7 items only assess the capacity for *Decentering*.

This new composite score was able to discriminate correctly between daily and non-experienced mindfulness practitioners in most cases (82%). Tools that can accurately discriminate between meditators and non-meditators, such as the MINDSENS index, are urgently needed in the field of mindfulness [17]. Unfortunately, most mindfulness measures are unable to differentiate between meditators and non-meditators [39] or report unexpected mindfulness levels when comparing control subjects and substance abusers [40].

When we used MINDSENS to explore the role of meditative practice in a sample population, we found that the two most relevant variables were related to practice (practice frequency and lifetime meditation experience). Surprisingly, however, the other two most relevant variables were sociodemographic (Years of schooling and Age), both of which were more closely associated with self-reported mindfulness than the length of meditation session. The importance of educational level on certain facets of mindfulness was previously reported by Baer et al. [6], who found that educational level was modestly correlated with all facets of FFMQ. Our data support this finding, since educational level was a predictive factor for MINDSENS and all but *Observing* and *Awareness* facets. In fact, *Describing* was associated only with educational level but not with any of the practice variables, a finding that can be interpreted as indicating that *Describing* has a weaker connection to the mindfulness experience. In terms of other socio-demographic variables, the variable Age had more influence than any practice variable on *Observation* and *Decentering*. We found no association between Age and *Awareness*, in contrast to Baer et al. [6]. It is possible that Age has a co-variation with the lifetime amount of practice, thus creating a potential analysis bias. Interestingly, *Awareness* was not explained by socio-demographic variables and only weakly by practice variables, a finding that raises the possibility that perhaps the remaining variance can be explained by dispositional factors [41], and this possibility suggests that these trait aspects may account for a significant proportion of the variance in other mindfulness facets, even in MINDSENS.

Mindfulness is a multifaceted construct that appears to be determined by different types of learning and training processes, including meditation and cognitive learning methods. In our study, we found that *Observing, Non-reactivity*, and *Decentering* all seem to be especially sensitive to cumulative rehearsal through formal meditation. However, sources of learning other than meditation may also have an impact on many of the facets and skills evaluated in this study. The mutual reinforcement between meditation practice and cognitive learning is not a new phenomenon, and the Buddhist tradition has long held that “intellectual comprehension […] reveals to be quite essential to ultimate success in the practice” [7]. So, even though meditators can learn, through meditation, that “thoughts are not facts”, they can also learn this in cognitive therapy [42], [33] or they can learn to be non-judgemental by following principles of radical acceptance in DBT [30]. As a result, it is possible to acquire such knowledge and understanding by means of psychoeducation and self-observation, neither of which require meditation practice. These alternative learning processes may influence facets such as *Describing* and *Non-judging* that are sensitive to education.

## Limitations

One possible limitation of the study is that the sample was recruited on the Internet. Despite the large sample size (n >500) and the existence of studies that confirm the reliability of data obtained from this source [43], these samples are probably more heterogeneous and biased–due to the high non-response rate–than those obtained by other, more traditional methods. In addition, as all the data was self-reported, the responses to the surveys may have been influenced by socially-desirable responses. Furthermore, frequency of meditation was measured by a recall system, and only present frequency was reported, not the consistency of practice. Another limitation is that we did not investigate any clinical outcomes related to mindfulness skills or amount of practice, and so we are unable make any inferences regarding the importance of our results for a clinical population. This issue is relevant and should be addressed in further studies. In addition, the literature refers to the relationship between home practice and changes in mindfulness traits in the context of mindfulness-based interventions, but in our study, practice was not understood as homework, but as an intentional practice, thus implying that the volunteer subjects in this study differed in many ways (motivational level, knowledge of meditation and moods, and personality aspects) compared to a sample recruited in the context of an interventional study. Although the effect of sex and age on mindfulness facets were taken into account in our analyses, the differences between groups in these variables could also be considered a limitation.

In spite of the aforementioned limitations, the findings from this study increase our knowledge on how meditation practice influences mindfulness and could assist trainers and researchers in designing more efficient mindfulness-based programs. Given that not all aspects of mindfulness are linked to meditation, we developed a new composite index that is more sensitive to practice variables in order to better measure the effects of practice. We believe this instrument–the MINDSENS index–could be useful in further research in the field of mindfulness.
